# Metabolic Score for Insulin Resistance and New-Onset Type 2 Diabetes in a Middle-Aged and Older Adult Population: Nationwide Prospective Cohort Study and Implications for Primary Care

**DOI:** 10.2196/49617

**Published:** 2024-06-03

**Authors:** Hui Cheng, Zhihui Jia, Yu Ting Li, Xiao Yu, Jia Ji Wang, Yao Jie Xie, Jose Hernandez, Harry H X Wang

**Affiliations:** 1 School of Public Health Sun Yat-Sen University Guangzhou China; 2 State Key Laboratory of Ophthalmology, Zhongshan Ophthalmic Center Sun Yat-Sen University Guangzhou China; 3 Guangdong Provincial Key Laboratory of Ophthalmology and Visual Science, Zhongshan Ophthalmic Center Sun Yat-Sen University Guangzhou China; 4 School of Public Health Guangzhou Medical University Guangzhou China; 5 School of Nursing The Hong Kong Polytechnic University Hung Hom, Kowloon China (Hong Kong); 6 Faculty of Medicine and Health EDU, Digital Education Holdings Ltd Kalkara Malta; 7 Green Templeton College University of Oxford Oxford United Kingdom; 8 Jockey Club School of Public Health and Primary Care, Faculty of Medicine The Chinese University of Hong Kong Shatin, New Territories China (Hong Kong); 9 Usher Institute, Deanery of Molecular, Genetic & Population Health Sciences The University of Edinburgh Edinburgh United Kingdom

**Keywords:** metabolic score for insulin resistance, type 2 diabetes mellitus, blood pressure, longitudinal study, primary care

## Abstract

**Background:**

The metabolic score for insulin resistance (METS-IR) has emerged as a noninsulin-based index for the approximation of insulin resistance (IR), yet longitudinal evidence supporting the utility of METS-IR in the primary prevention of type 2 diabetes mellitus (T2DM) remains limited.

**Objective:**

We aimed to investigate the longitudinal association between METS-IR, which combines fasting plasma glucose (FPG), lipid profiles, and anthropometrics that can be routinely obtained in resource-limited primary care settings, and the incidence of new-onset T2DM.

**Methods:**

We conducted a closed-cohort analysis of a nationwide, prospective cohort of 7583 Chinese middle-aged and older adults who were free of T2DM at baseline, sampled from 28 out of 31 provinces in China. We examined the characteristics of participants stratified by elevated blood pressure (BP) at baseline and new-onset T2DM at follow-up. We performed Cox proportional hazard regression analysis to explore associations of baseline METS-IR with incident T2DM in participants overall and in participants stratified by baseline BP. We also applied net reclassification improvement and integrated discrimination improvement to examine the incremental value of METS-IR.

**Results:**

During a mean follow-up period of 6.3 years, T2DM occurred in 527 participants, among which two-thirds (332/527, 62.9%; 95% CI 58.7%-67.1%) had baseline FPG<110 mg/dL. A SD unit increase in baseline METS-IR was associated with the first incidence of T2DM (adjusted hazard ratio [aHR] 1.33, 95% CI 1.22-1.45; *P*<.001) in all participants. We obtained similar results in participants with normal baseline BP (aHR 1.41, 95% CI 1.22-1.62; *P*<.001) and elevated baseline BP (aHR 1.29, 95% CI 1.16-1.44; *P*<.001). The predictive capability for incident T2DM was improved by adding METS-IR to FPG. In study participants with new-onset T2DM whose baseline FPG was <126 mg/dL and <110 mg/dL, 62.9% (332/527; 95% CI 60%-65.9%) and 58.1% (193/332; 95% CI 54.3%-61.9%) of participants had baseline METS-IR above the cutoff values, respectively.

**Conclusions:**

METS-IR was significantly associated with new-onset T2DM, regardless of baseline BP level. Regular monitoring of METS-IR on top of routine blood glucose in clinical practice may add to the ability to enhance the early identification of primary care populations at risk for T2DM.

## Introduction

Type 2 diabetes mellitus (T2DM) exerts a profound impact on the health and well-being of individuals, families, and communities worldwide because of its high prevalence, concomitant increase in risks of complications and treatment costs, and reduced quality of life [1-4]. Data from the European Association for the Study of Diabetes, the American Diabetes Association, and the International Diabetes Federation consistently indicate a rising epidemic of T2DM regionally and globally [5,6]. In low- and middle-income countries, the rapid progress of urbanization, aging, lifestyle transformations, and a lack of sustainable health education have further posed significant challenges to the prevention of T2DM [6-8]. Moreover, patients at older ages with poor glycemic control may suffer from progressive pathological as well as functional decline [9]. Evidence suggests that the progression of T2DM and its complications can largely be delayed through population-based early preventive public health programs [10,11].

Insulin resistance (IR) is a common feature of prediabetes and prehypertension and also a precursor to the development of both conditions [12]. Recommendations stress that the initiation of intervention during the stage of IR, instead of after a T2DM diagnosis, is more effective in reducing treatment burden and overall health care costs [13]. The hyperinsulinemic-euglycemic clamp (HEC) has been widely considered the “gold standard” experimental method for direct determination of IR, although less popular in daily practice due to its invasiveness, expensiveness, and complex procedures [14]. The homeostatic model assessment for IR and the quantitative insulin sensitivity check index, which are both fasting insulin-based indexes [15], are also less commonly performed as part of regular checkups in low- and middle-income countries’ primary care settings.

The metabolic score for insulin resistance (METS-IR), a novel index that considers fasting plasma glucose (FPG), lipid profiles, and an obesity index that can be easily obtained during routine examination, has been proposed as a simple and high-accuracy measure to assess IR in the western population [14]. Evaluation of METS-IR may reduce the cost associated with the immunoassay of insulin and the heterogeneity between different analytical methods [14,16]. The hypothesis that METS-IR is predictive of new-onset T2DM over time needs further testing in the Chinese middle-aged and older adult population, which accounts for 42.2% of the total population [17]. From a multimorbidity perspective, whether the association between METS-IR and first-incident T2DM is persistent among participants whose blood pressure (BP) falls within the normal range also remains uncertain.

We aimed to investigate the association between METS-IR at baseline and the incidence of new-onset T2DM at follow-up in a middle-aged and older adult population. We also sought to examine whether the predictive capability of METS-IR on top of routine blood glucose for incident T2DM differed between participants with and those without elevated baseline BP.

## Methods

### Study Design

We conducted a cohort analysis of nationally representative survey data retrieved from the China Health and Retirement Longitudinal Study between 2011 and 2018, run by Peking University in partnership with the National Natural Science Foundation of China, the National Institute on Aging, and the World Bank. The data collected during the study period are made available in the public domain. The China Health and Retirement Longitudinal Study was originally designed after the US Health and Retirement Study and other internationally developed aging-related surveys to collect high-quality data on various aspects of health and social care for middle-aged and older adults in China [18-21].

### Setting and Data Source

The nationwide baseline survey commenced in 2011 (wave 1). Follow-up surveys were repeatedly conducted in 2013 (wave 2), 2015 (wave 3), and 2018 (wave 4). The details of the sampling frame and household interview procedures were described elsewhere [18,19]. In brief, multistage, region-stratified, probability proportional to size sampling was adopted for sample selection from 150 county-level districts in 28 out of a total of 31 provinces across China. The baseline participants were drawn from over 10 thousand households in 450 neighborhood- and village-level units. Information on the individual’s sociodemographics, lifestyles, and health status was collected through face-to-face computer-assisted interviews in each wave. Data on anthropometric and clinical parameters were obtained by physical examinations, including blood drawing, for subsequent laboratory tests in waves 1 and 3 [19-21].

### Participants

A total of 17,708 participants were enrolled at baseline, of whom 17,314 were aged 45 years and older. We excluded individuals who had any of the following: (1) a clinical diagnosis of T2DM, FPG≥126 mg/dL (7 mmol/L), or glycated hemoglobin A_1c_ (HbA_1c_) ≥6.5% (n=2414); (2) missing information on the diagnosis of T2DM (n=130); (3) unknown BP levels (n=2490); (4) incomplete data on BMI, FPG, triglycerides, and high-density lipoprotein cholesterol (HDL-C) to calculate baseline METS-IR (n=4454); and (5) nonattendance at any of the follow-ups (n=243). This yielded a total of 7583 participants who met the eligibility criteria and were included in the closed-cohort analysis (Figure S1 in Multimedia Appendix 1).

### Study Variables and Measurements

Information on age, sex, place of residence, education level, household income, and living relationships was collected by centrally trained interviewers with an internationally comparable, validated survey instrument [19]. Self-reported lifestyles included current cigarette smoking and engagement in alcohol drinking at least once per month regularly. Anthropometric parameters were measured with participants in light clothing and without shoes, using a portable stadiometer (Seca 213 stadiometer [Seca Trading]) and a calibrated digital scale (Omron HN-286 scale [Krell Precision]) [19]. BMI was calculated as weight in kilograms divided by the square of height in meters (kg/m^2^). The BP of the arm with a higher value was measured in a seated position using a routinely validated automatic BP monitor (Omron HEM-7112/7200 monitor [Omron]), and the average of 3 BP readings taken at 45-second intervals was recorded [19]. A venous blood sample at fasting was collected for blood-based bioassays according to standard operating procedures [20,21]. The Hexokinase method was used for FPG measurement (mg/dL), while lipid panel profiles including total cholesterol (mg/dL) and triglycerides (mg/dL) were measured using the Oxidase method, and low-density lipoprotein cholesterol (mg/dL) and HDL-C (mg/dL) were determined using the direct method [21]. Glucose and lipid parameters, together with BMI, were taken into account in the assessment of METS-IR, which was calculated as: Ln((2×FPG)+triglyceride)×BMI/Ln(HDL-C) [14]. Elevated BP at baseline was defined as systolic BP≥120 mm Hg or diastolic BP≥80 mm Hg (or both) or the presence of physician-diagnosed hypertension [22]. This included both hypertension and high normal BP. The primary outcome of this study was the first incidence of physician-diagnosed T2DM during follow-up, which was double-verified using the information documented in the previous follow-up wave. Participants enrolled at baseline were followed until they had newly diagnosed T2DM or the recorded attendance at the most recent wave of follow-up, whichever came first.

### Statistical Analysis

Data are presented as n (%) values for categorical variables and as mean (SD) or median (IQR; 25th to 75th percentiles) values where appropriate for continuous variables. We examined the characteristics of participants stratified by the presence of elevated BP at baseline and new-onset T2DM at follow-up. The 2-tailed 2-sample *t* test, the nonparametric Wilcoxon rank sum test, or the chi-square test, where appropriate, was used for between-group comparisons in participants with and those without new-onset T2DM. Participants were further divided into quartiles of METS-IR, where the lowest quartile was used as the reference group. The cumulative hazard of T2DM was determined by the Kaplan-Meier plot, and the 2-sided logrank test was used for the overall comparison of curves across METS-IR quartiles. Cox proportional hazard regression models were constructed to estimate the risk of new-onset T2DM in participants overall and in participants stratified by baseline BP after adjusting for known important covariates, including demographics, socioeconomic status, lifestyles, and anthropometric measurements. The proportional hazards assumption for model fit was tested using the scaled Schoenfeld residuals. The adjusted hazard ratios (aHR) with a 95% CI were estimated for a unit increase in METS-IR per SD and for each METS-IR quartile. We further modeled the data as restricted cubic splines with 4 knots, located at the 5th, 35th, 65th, and 95th percentiles following the Akaike information criterion [23], of METS-IR to assess the shape of the association between METS-IR and the risk of T2DM. All models were adjusted for confounders included in the Cox models.

We also assessed the predictive capability of baseline METS-IR for new-onset T2DM. We applied net reclassification improvement and integrated discrimination improvement [24] to examine whether adding METS-IR to FPG may have incremental value in improving the predictive accuracy for T2DM. Furthermore, the optimal cutoff points with the highest Youden index were derived from the receiver operating characteristic (ROC) analysis. We calculated the proportion of people with new-onset T2DM who had baseline METS-IR above the corresponding cutoff value in all participants and in the subgroup without baseline impaired fasting glucose (ie, FPG<110 mg/dL [25]).

A series of sensitivity analyses were performed to ensure the reliability of the results. We repeated the aforementioned Cox models fitted in the main analysis under 3 different scenarios. First, participants who had an incident of T2DM at the first follow-up visit were excluded to account for possible reverse causality bias. Second, participants with the presence of general obesity (ie, BMI ≥28 kg/m^2^ according to the Working Group on Obesity in China [26]) at baseline were excluded given the strong association between obesity and incident diabetes. Third, we used laboratory test results of FPG and HbA_1c_, where available, to supplement the information on the occurrence of T2DM (ie, FPG≥126 mg/dL [7 mmol/L] or HbA_1c_ ≥6.5%) to ascertain whether associations between METS-IR and T2DM may vary from the main analysis. We also treated age, socioeconomic status, lifestyles, and anthropometric measurements as time-dependent variables in the Cox models built in the main analysis to take into account the potential time-varying effects of covariates. In addition, the demographic characteristics of participants included in the analysis were compared with those of participants excluded due to missing values to understand the potential selection bias and its impact on the generalizability of the findings. Analyses were conducted using SAS (version 9.4; SAS Institute Inc) and R (version 4.0.2; R Core Team). A 2-tailed *P* value of less than .05 was considered statistically significant.

### Ethical Considerations

All study participants provided written consent at enrolment. Data are publicly archived by Peking University, where ethics approval was obtained (IRB00001052-11014 and IRB00001052-11015). The ethics of the present analysis were sought from the Biomedical Research Ethics Review Committee at Sun Yat-Sen University (SPH2019123). Data anonymization was performed by removing all participant identifiers before data analysis.

## Results

### Study Population and Baseline Characteristics

Data were analyzed among a total of 7583 participants free of T2DM at baseline (Figure S1 in Multimedia Appendix 1). Participants ranged in age between 45 and 96 years, with a mean age of 59.1 (SD 9.3) years, sampled from 28 out of 31 provinces. Around one-third (2502/7583) of participants had a BP<120/80 mm Hg at baseline. T2DM occurred in 527 participants (ie, 214 male participants and 313 female participants) over a mean of 6.3 years of follow-up, among which nearly two-thirds (332/527, 62.9%; 95% CI 58.7%-67.1%) had baseline FPG<110 mg/dL (6.1 mmol/L). The baseline METS-IR of participants who had T2DM at follow-up was significantly higher than that of nonincident T2DM counterparts (36.1, SD 8.4 vs 32.6, SD 6.6; *P*<.001 among those with normal baseline BP and 38.9, SD 8 vs 35.3, SD 7.8; *P*<.001 among those with elevated baseline BP; Table 1). In participants with normal baseline BP, those who developed T2DM had significantly higher levels of BMI, waist circumference, FPG, and triglyceride but lower HDL-C at baseline when compared to those free of new-onset T2DM. A similar pattern was observed in participants with elevated baseline BP (Table S1 in Multimedia Appendix 1). Relative to participants who were excluded due to missing information (METS-IR, BP, FPG, and lost to follow-up), this study sample had a greater proportion of female participants (4051/7583, 53.4% vs 3572/7317, 48.8%), rural residents (5029/7583, 66.3% vs 3948/7317, 54%), those with lower education level (5325/7583, 70.2% vs 4558/7317, 62.5%), and current smokers (2317/7583, 30.7% vs 1908/7317, 28.7%), despite similar mean age (59.1 vs 59.2 years; *P*=.84) and alcohol drinking profile (2522/7583, 33.3% vs 2478/7317, 34.4%; *P*=.15; Table S2 in Multimedia Appendix 1).

The 2-sample *t* test or the chi-square test, where appropriate, was used for between-group comparisons among participants with and those without new-onset T2DM (ie, T2DM at follow-up vs non-T2DM at follow-up). Normal BP was defined as systolic BP<120 mm Hg and diastolic BP<80 mm Hg. Elevated BP was defined as systolic BP≥120 mm Hg or diastolic BP≥80 mm Hg (or both) or the presence of physician-diagnosed hypertension according to the 2018 Chinese Guidelines for the Management of Hypertension.

**Table 1:** Characteristics of participants by blood pressure (BP) at baseline and new-onset type 2 diabetes mellitus (T2DM) at follow-up.

Characteristics							Normal BP at baseline			Elevated BP at baseline		
							T2DM at follow-up (n=116)	Non-T2DM at follow-up (n=2386)	*P* value	T2DM at follow-up (n=411)	Non-T2DM at follow-up (n=4670)	*P* value
**Age groups (years), n (%)**									.10			.03
						45-54	40 (34.5)	1052 (44.1)		135 (32.8)	1390 (29.8)	
						55-64	50 (43.1)	928 (38.9)		171 (41.6)	1793 (38.4)	
						≥65	26 (22.4)	406 (17)		105 (25.6)	1487 (31.8)	
**Sex, n (%)**									.35			.004
						Male	47 (40.5)	1073 (44.97)		167 (40.6)	2245 (48.1)	
						Female	69 (59.5)	1313 (55.03)		244 (59.4)	2425 (51.9)	
**Place of residence, n (%)**									.21			.62
						Urban	30 (25.9)	749 (31.4)		139 (33.8)	1636 (35)	
						Rural	86 (74.1)	1637 (68.6)		272 (66.2)	3034 (65)	
**Education level, n (%)**									.07			.74
						Elementary school or below	88 (75.9)	1612 (67.6)		290 (70.7)	3335 (71.4)	
						Middle school or above	28 (24.1)	774 (32.4)		120 (29.3)	1335 (28.6)	
**Annual household income, n (%)**									.36			.76
						Quartile 1^a^ (most deprived)	23 (20.2)	502 (21.2)		110 (27)	1250 (26.9)	
						Quartile 2^b^	36 (31.6)	588 (24.8)		103 (25.25)	1161 (25)	
						Quartile 3^c^	30 (26.3)	630 (26.6)		105 (25.7)	1112 (24)	
						Quartile 4^d^ (most affluent)	25 (21.9)	648 (27.4)		90 (22.1)	1120 (24.1)	
**Living relationships, n (%)**									.67			.52
						Living with a partner	103 (88.8)	2087 (87.4)		341 (83)	3815 (81.7)	
						Living alone	13 (11.2)	299 (12.5)		70 (17)	855 (18.3)	
**Cigarette smoking, n (%)**									.93			<.001
						Current smoker	35 (30.2)	726 (30.6)		92 (22.5)	1464 (31.5)	
						Nonsmoker	81 (69.8)	1650 (69.4)		317 (77.5)	3190 (68.5)	
**Alcohol drinking, n (%)**									.60			.001
						Regular drinker	36 (31)	796 (33.4)		107 (26)	1583 (33.9)	
						Nondrinker	80 (69)	1588 (66.6)		304 (74)	3084 (66.1)	
METS-IR^e^ index, mean (SD)							36.1 (8.4)	32.6 (6.6)	<.001	38.9 (8)	35.3 (7.8)	<.001

^a^Quartile 1: ≤CNY 5200 (≤US $719.79).

^b^Quartile 2: ≥CNY 5201 to CNY 15,300 (≥US $719.93-$2117.85).

^c^Quartile 3: ≥CNY 15,301 to CNY 34,176 (≥US $2117.99-$4730.70).

^d^Quartile 4: ≥CNY 34,177 (≥US $4730.84).

^e^METS-IR: metabolic score for insulin resistance.

### The First Incidence of Physician-Diagnosed T2DM at Follow-Up

**Figure 1:**
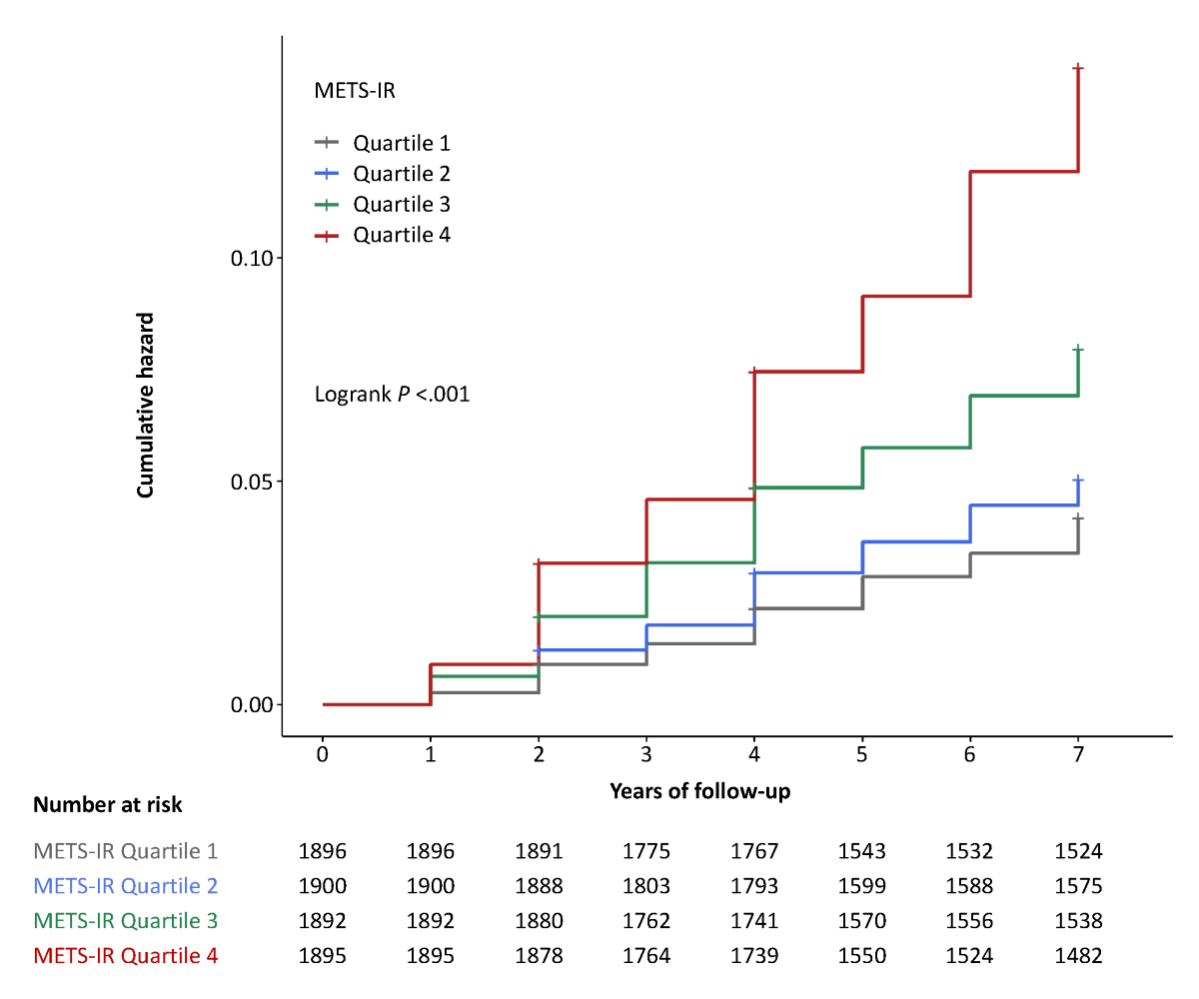
Cumulative hazard of type 2 diabetes mellitus by quartiles of metabolic score for insulin resistance (METS-IR) at baseline. Quartile 1: ≤29.18; Quartile 2: ≥29.19 to 33.47; Quartile 3: ≥33.48 to 38.97; and Quartile 4: ≥38.98.

The incidence of new-onset T2DM was reached at a rate of 11 per 1000 person-years of follow-up in overall participants (ie, 7.2 and 13 per 1000 person-years for participants with normal baseline BP and elevated baseline BP, respectively; Table 2). The highest cumulative hazard of T2DM was observed at 7 years in participants with the highest quartile of baseline METS-IR (13.3%) when compared to that in those with the lowest quartile (4.1%), with a difference of 9.2 percentage points (95% CI 7.4-11; Figure 1).

**Table 2:** Incidence rate of physician-diagnosed type 2 diabetes mellitus (T2DM) at follow-up and associations between baseline metabolic score for insulin resistance (METS-IR) and new-onset T2DM.

Variables				T2DM cases, n	Incidence rate^a^		Crude model		Adjusted model			
							HR^b^ (95% CI)	*P* value	aHR^c^ (95% CI)		*P* value	
**METS-IR, per SD unit increase**												
	All participants at baseline			527	11		1.44 (1.35-1.53)	<.001	1.33 (1.22-1.45)		<.001	
	Participants with normal blood pressure at baseline			116	7.2		1.38 (1.23-1.54)	<.001	1.41 (1.22-1.62)		<.001	
	Participants with elevated blood pressure at baseline			411	13		1.41 (1.31-1.52)	<.001	1.29 (1.16-1.44)		<.001	
**METS-IR, quartiles**												
	**All participants at baseline**											
		Quartile 1 (≤29.18)		70	5.9		1 (Reference)	N/A^d^	1 (Reference)		N/A	
		Quartile 2 (29.19-33.47)		87	7.2		1.22 (0.89-1.67)	.22	1.16 (0.84-1.6)		.37	
		Quartile 3 (33.48-38.97)		135	11.3		1.93 (1.44-2.57)	<.001	1.71 (1.25-2.34)		<.001	
		Quartile 4 (≥38.98)		235	19.9		3.39 (2.6-4.43)	<.001	2.72 (1.95-3.79)		<.001	
		*P* value for trend		N/A	N/A		1.08 (1.07-1.1)	<.001	1.07 (1.05-1.09)		<.001	
	**Participants with normal blood pressure at baseline**											
		Quartile 1 (≤28.22)		22	5.5		1 (Reference)	N/A	1 (Reference)		N/A	
		Quartile 2 (28.23-31.7)		13	3.2		0.59 (0.3-1.16)	.13	0.64 (0.32-1.28)		.20	
		Quartile 3 (31.71-36.18)		33	8.1		1.48 (0.86-2.53)	.16	1.59 (0.88-2.87)		.13	
		Quartile 4 (≥36.19)		48	12.1		2.22 (1.34-3.68)	.002	2.29 (1.21-4.33)		.011	
		*P* for trend		N/A	N/A		1.08 (1.04-1.12)	<.001	1.08 (1.03-1,12)		<.001	
	**Participants with elevated blood pressure at baseline**											
		Quartile 1 (≤29.87)		55	7.0		1 (Reference)	N/A	1 (Reference)		N/A	
		Quartile 2 (29.88-34.59)		70	8.8		1.25 (0.88-1.77)	.22	1.16 (0.81-1.66)		.44	
		Quartile 3 (34.6-40.26)		110	13.9		1.98 (1.43-2.73)	<.001	1.7 (1.2-2.42)		.003	
		Quartile 4 (≥40.27)		176	22.2		3.17 (2.34-4.28)	<.001	2.52 (1.73-3.67)		<.001	
		*P* for trend		N/A	N/A		1.07 (1.06-1.09)	<.001	1.06 (1.04-1.08)		<.001	

^a^per 1000 person-years.

^b^HR: hazard ratio.

^c^aHR: adjusted hazard ratio.

^d^Not applicable.

Normal BP at baseline was defined as systolic BP<120 mm Hg and diastolic BP<80 mm Hg. Elevated BP at baseline was defined as systolic BP≥120 mm Hg or diastolic BP≥80 mm Hg (or both) or the presence of physician-diagnosed hypertension according to the 2018 Chinese Guidelines for the Management of Hypertension. Cox proportional hazard model adjusted for age, sex, place of residence, education level, annual household income, living relationships, cigarette smoking, alcohol drinking, BP, and waist circumference. Tests for trend are based on variables containing the median value for each quartile.

### Associations Between Baseline METS-IR and First Incident T2DM

**Figure 2:**
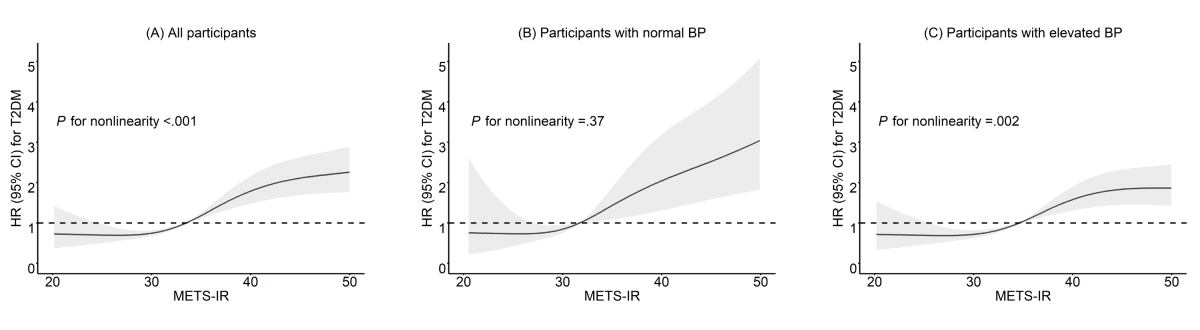
Restricted cubic spline estimates of the relationship between the metabolic score for insulin resistance (METS-IR) and new-onset type 2 diabetes mellitus (T2DM). BP: blood pressure; HR: hazard ratio.

An SD unit increase in baseline METS-IR was associated with the first incidence of T2DM (aHR 1.32, 95% CI 1.22-1.45; *P*<.001) in all participants. The adjusted risk of new-onset T2DM in participants with the highest quartile of baseline METS-IR was 2.72-fold higher than that in those with the lowest quartile of METS-IR. Similar trends were obtained from both crude and adjusted models, irrespective of baseline BP levels (Table 2). The 4-knot curve demonstrated a nonlinear association between baseline METS-IR and new-onset T2DM in participants overall and in participants with elevated baseline BP (Figure 2). The same models were repeated in the sensitivity analyses with consistent results (Tables S3-S6 in Multimedia Appendix 1).

Restricted cubic splines with 4 knots, located at the 5th, 35th, 65th, and 95th percentiles of METS-IR for (A) all participants, (B) participants with normal BP, and (C) participants with elevated BP. The solid black line represents the fitted curve, and the gray bands represent the 95% CI bands. The model was adjusted for age, sex, place of residence, education level, annual household income, marital relationship, cigarette smoking, alcohol drinking, BP, and waist circumference. Normal BP was defined as systolic BP<120 mm Hg and diastolic BP<80 mm Hg. Elevated BP was defined as systolic BP≥120 mm Hg or diastolic BP≥80 mm Hg (or both) or the presence of physician-diagnosed hypertension according to the 2018 Chinese Guidelines for the Management of Hypertension.

### The Predictive Capability of Baseline METS-IR for New-Onset T2DM

**Figure 3:**
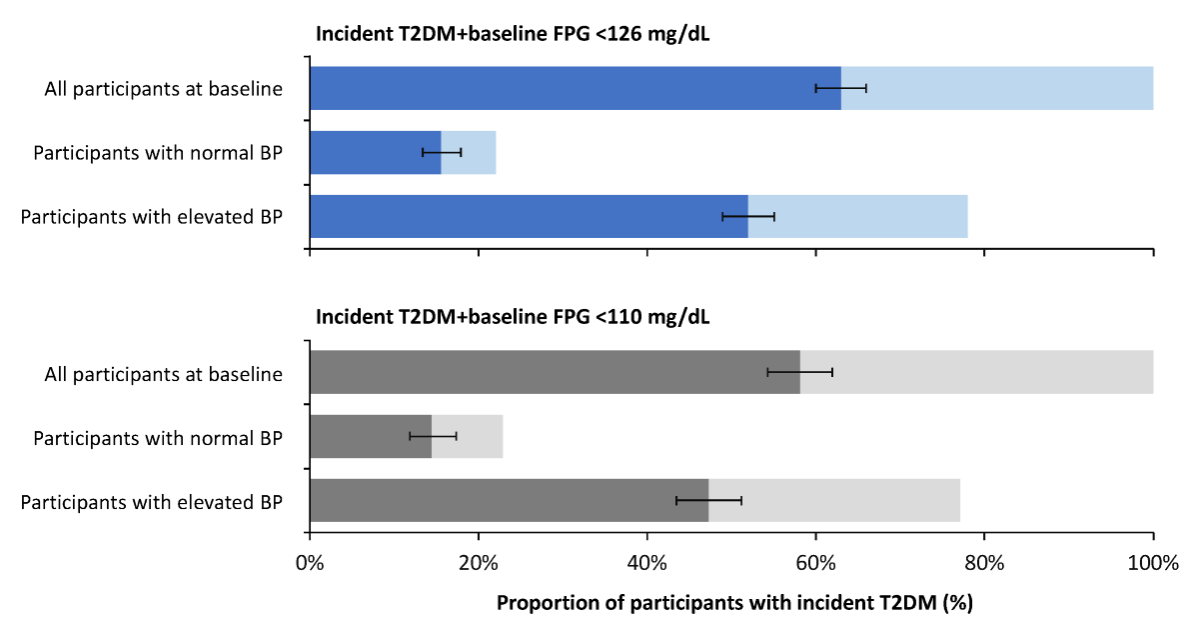
Proportion of participants with new-onset type 2 diabetes mellitus (T2DM) at follow-up by blood pressure (BP) at baseline. FPG: fasting plasma glucose.

The ROC curves based on the predicted probabilities for incident T2DM showed that the area under the curve ranged from 0.633 to 0.708 across all models. Both net reclassification improvement and integrated discrimination improvement for predicting new-onset T2DM increased by adding METS-IR to FPG, irrespective of baseline elevated BP (Table S7 in Multimedia Appendix 1). In all participants with new-onset T2DM whose baseline FPG levels were <126 mg/dL (7 mmol/L) and <110 mg/dL (6.1 mmol/L), 62.9% (332/527; 95% CI 60%-65.9%) and 58.1% (193/332; 95% CI 54.3%-61.9%) had baseline METS-IR above cutoff values (ie, ≥35.3 for all participants, ≥31.7 for participants with normal BP, and ≥35.5 for participants with elevated BP), respectively. The proportion of participants with baseline METS-IR above the corresponding cutoff values was similar between participants with and those without elevated baseline BP (Figure 3).

The total length of each bar represents the share of participants with new-onset T2DM at follow-up who had baseline FPG<110 mg/dL (6.1 mmol/L) and baseline FPG<126 mg/dL (7 mmol/L), respectively. The darker bars represent the share of participants with new-onset T2DM at follow-up who had baseline METS-IR above the estimated threshold (ie, ≥35.3 for all participants, ≥31.7 for participants with normal BP, and ≥35.5 for participants with elevated BP), with thin bars representing 95% CIs. To convert glucose values from mg/dL to mmol/L, multiply by 0.055.

## Discussion

### Main Findings

We provided longitudinal evidence supporting the potential utility of METS-IR in the prediction of T2DM in a large, nationally representative sample of the Chinese middle-aged and older adult population. We demonstrated significant associations between increased baseline METS-IR and a higher incidence of T2DM, regardless of the presence of baseline elevated BP. The predictive capability for incident T2DM was improved by adding METS-IR to FPG. In participants with incident T2DM whose baseline FPG fell within the normal range, around two-thirds had baseline METS-IR above the cutoff values, implying that the use of METS-IR on top of routine blood glucose in clinical practice may add additional value to early identification of populations at risk for T2DM.

### Relationship With Other Studies

The role that METS-IR plays as a complement to previously validated risk prediction models is an emerging area of research interest. As a novel noninsulin-based index for estimating insulin sensitivity, METS-IR combines fasting laboratory values and anthropometric measurements that are routinely available in primary care instead of relying on insulin-based measurements that are laborious, expensive, and of high variability due to different immunoassay techniques [16]. Recent evidence indicates a satisfactory validity of METS-IR against the adjusted whole-body glucose disposal rate (ie, M value) derived from the “gold standard” HEC and the reliability of METS-IR when compared to both fasting insulin-based indexes (eg, homeostatic model assessment for IR and quantitative insulin sensitivity check index) and noninsulin-based indexes (eg, triglyceride-glucose index, triglyceride-glucose-BMI index, and triglyceride/HDL-C ratio) for predicting T2DM in a Mexican outpatient cohort [14]. The relationship between METS-IR and the occurrence of T2DM observed in the Latin-American population was similarly documented in subsequent studies among nonobese adults recruited in the Japanese NAfld in the Gifu Area, Longitudinal Analysis cohort [27,28] and among rural adults in Henan, northern China [29]. Further to our previous cross-sectional investigation in an urbanized township in southern China [30], we extended the analysis by using a large cohort of middle-aged and older adults that are nationally representative, while taking into account the urban-rural heterogeneity in lifestyles, income, and education profiles, as well as health care disparities that are prevailing in developing countries [31]. We found a consistent relationship between increased baseline METS-IR and risks for new-onset T2DM, irrespective of BP levels, stressing that normotensive people should not be neglected in diabetes prevention.

The potential association between METS-IR and incident T2DM may have several mechanisms. METS-IR was found to be significantly correlated with visceral, intrahepatic, and intrapancreatic fat content that may contribute to pathophysiological alterations of glucose and lipid homeostasis [14,32-35]. This may translate into chronically elevated levels of glucose and fatty acids, thereby inducing toxic states in pancreatic islets and progressive worsening of beta-cell function due to glucotoxicity, lipotoxicity, and glucolipotoxicity [36]. In addition to plasma triglyceride and HDL‐C that are related to insulin‐mediated glucose disposal, the inclusion of BMI as a surrogate for visceral adiposity takes into account the causal relationship between adipose tissue inflammation and the development of IR and ultimately T2DM [37-39]. The nonlinear relationship we observed between baseline METS-IR and new-onset T2DM might be partly explained by the multifactorial interactions among adiposity, dysglycemia, and inflammation in metabolic pathways and vascular biologic processes [13,39-42]. Our findings echo previous literature on the nonlinear relationship between FPG and T2DM in the European population [43]. Given that splines were constructed from piecewise polynomials, the shape of the restricted cubic splines curve could be largely influenced by the location and number of knots. It is also worth noting that the cutoff values derived from the ROC analysis should not be interpreted as a rigid diagnostic threshold but rather as a reference level above which regular monitoring of METS-IR would probably yield additional value in targeted interventions despite normal blood glucose and therefore may be meaningful for risk assessment and risk communication in diabetes prevention. The use of METS-IR has also been extended to T2DM-related cardiovascular conditions in Mexican and South Korean populations [44,45], indicating the potential for wider applicability in community-based practice where a valid, easy-to-measure, and less resource-consuming prediction tool is preferable.

### Strengths and Weaknesses of the Study

We examined the longitudinal relationships of baseline METS-IR and first incident T2DM in a large, nationally representative sample while evaluating potential nonlinear associations. The analyses were systematically performed using METS-IR as a continuous variable and in quartiles, with a consistent methodology adopted to deal with confounding and reverse causality. A fairly extensive range of sensitivity analyses made little difference in the estimated associations between baseline METS-IR and new-onset T2DM. A particular strength was that the size of the study allowed us to retain power while stratifying participants by the presence of baseline elevated BP and thus considering the challenge of addressing the most common modality of multimorbidity encountered in the real-world clinical setting. This study has some possible limitations that merit consideration. First, the Cox models were built on a specific cohort of population samples who were middle-aged and older adults, and thus the generalizability of our findings to a wider population, or patients in certain health care settings, should be interpreted with caution. Selection bias may occur as nearly half of participants from the source cohort were not included in the present analysis given the eligibility criteria, although comparable in population mean age and drinking profile. Second, the HEC was not available for direct assessment of insulin sensitivity due to time and budget constraints, which may inevitably affect the accuracy of the assessment. However, complex and expensive procedures for determining insulin levels may limit implementation feasibility in large-scale studies in low-resource settings. Third, undiagnosed diabetes at baseline might have led to an underestimation of hazard ratios for new-onset T2DM. Fourth, we were not able to take account of the unmeasured confounders such as dietary regimes [46], body constitutions [47], environmental exposures, and health care usage that may exert cohort effects. We also acknowledge that time-varying confounding is impossible to fully overcome given that past exposures were not adequately captured. Fifth, we cannot rule out the possibility of random measurement errors in a large-scale multisite study, albeit with a standardized procedure for data collection. Last but not least, the blood drawing was voluntary and not performed in waves 2 and 4, and thus we were unable to cross-check the incidence of physician-diagnosed T2DM based on FPG and HbA_1c_. Nevertheless, the supplement use of biomedical records where available at follow-up in the sensitivity analyses yielded consistent results, suggesting the robustness of our findings.

### Implications for Research and Clinical Practice

This study carries a clinically meaningful message for primary care physicians with a potentially greater opportunity for recognition of middle-aged and older adults early in the course of their diabetes. Physicians working in collaboration with other health care professionals can help those at-risk individuals enhance their capacity to optimize blood glucose, BP, BMI, and lipid profiles through health education paired with effective strategies such as lifestyle coaching and skill building. This is in line with recent recommendations from the United States and Europe, advocating that early and sustainable preventative efforts could spare the expense of managing T2DM and its long-term complications [48,49]. Middle-aged and older adults whose blood glucose falls within the normal range but have a higher METS-IR might remain at risk for T2DM and thus should still be monitored for disease occurrence through regular follow-up along with individualized risk assessment. This also provides an impetus for future studies to explore whether the biological underpinnings of METS-IR-T2DM may vary among individuals with different phenotypes of visceral obesity and genetic predispositions. The age of 45 years was used as a cutoff for middle age, which is in line with the eligibility criteria widely adopted in other large-scale population-based cohort studies such as the Healthy, Aging, and Retirement in Thailand Study [50], the Swedish EpiHealth Study [51], and the Canadian Longitudinal Study on Aging [52]. The Rotterdam Study, which was originally comprised of participants aged 45 years or older, has expanded the cohort that targeted participants aged 40 years or older since 2016 [53]. This echoes the shift toward earlier onset of T2DM [54,55], and thus future pragmatic trials to evaluate the use of METS-IR may be extended to the younger population. Alongside the ongoing efforts to translate team-based care into practice on a global scale [56], further steps to integrate the monitoring of METS-IR, rather than blood glucose alone, into the existing approach to diabetes prevention are especially promising. This would help inform broad public health policy and community-oriented preventive strategies that include early identification of individuals at risk of T2DM through innovative surveillance tools based on readily available routine physical examination indicators.

In conclusion, METS-IR was significantly associated with new-onset T2DM, regardless of baseline BP level. Regular monitoring of METS-IR on top of routine blood glucose in resource-limited settings may add to the ability to enhance early recognition and appropriate management of individuals at risk for T2DM in primary care.

## Appendix

Multimedia Appendix 1 Diagram of study flow; Clinical parameters of participants by BP at baseline and new-onset T2DM at follow-up; Comparison of demographic characteristics between participants included in the final analysis and those excluded due to missing values; Sensitivity analyses; Predictive capability of baseline METS-IR on top of blood glucose for new-onset T2DM.

## Abbreviations

aHR: adjusted hazard ratio
BP: blood pressure
FPG: fasting plasma glucose
HbA_1c_: hemoglobin A_1c_
HDL-C: high-density lipoprotein cholesterol
HEC: hyperinsulinemic-euglycemic clamp
IR: insulin resistance
METS-IR: metabolic score for insulin resistance
ROC: receiver operating characteristic
T2DM: type 2 diabetes mellitus

## Data Availability

The original China Health and Retirement Longitudinal Study data were made available in the public domain by Peking University. The data sets generated or analyzed during the current study are available from the corresponding author on reasonable request.
