# Supplementary material for: Metabolic Score for Insulin Resistance and New-Onset Type 2 Diabetes in a Middle-Aged and Older Adult Population: Nationwide Prospective Cohort Study and Implications for Primary Care
Source: JMIR Public Health Surveill. 2024 Jun 3;10:e49617. doi: 10.2196/49617 (PMC11184265; doi:10.2196/49617)
Supplement: Multimedia Appendix 1 [file publichealth_v10i1e49617_app1.docx]

**Multimedia Appendix**

[JPH ms#49617]

**Figure S1** Diagram of study flow

**Table S1** Clinical parameters of participants by BP at baseline and new-onset T2DM at follow-up

**Table S2** Comparison of demographic characteristics between participants included in the final analysis and those excluded due to missing values

**Table S3** Sensitivity analysis by excluding participants who had new-onset T2DM within the first follow-up visit

**Table S4** Sensitivity analysis by excluding participants with general obesity at baseline

**Table S5** Sensitivity analysis by supplementing incidence data using laboratory blood test results where data were available

**Table S6** Sensitivity analysis based on models fitted with time-varying covariates

**Table S7** Predictive capability of baseline METS-IR on top of blood glucose for new-onset T2DM

**Figure S1 Diagram of study flow**


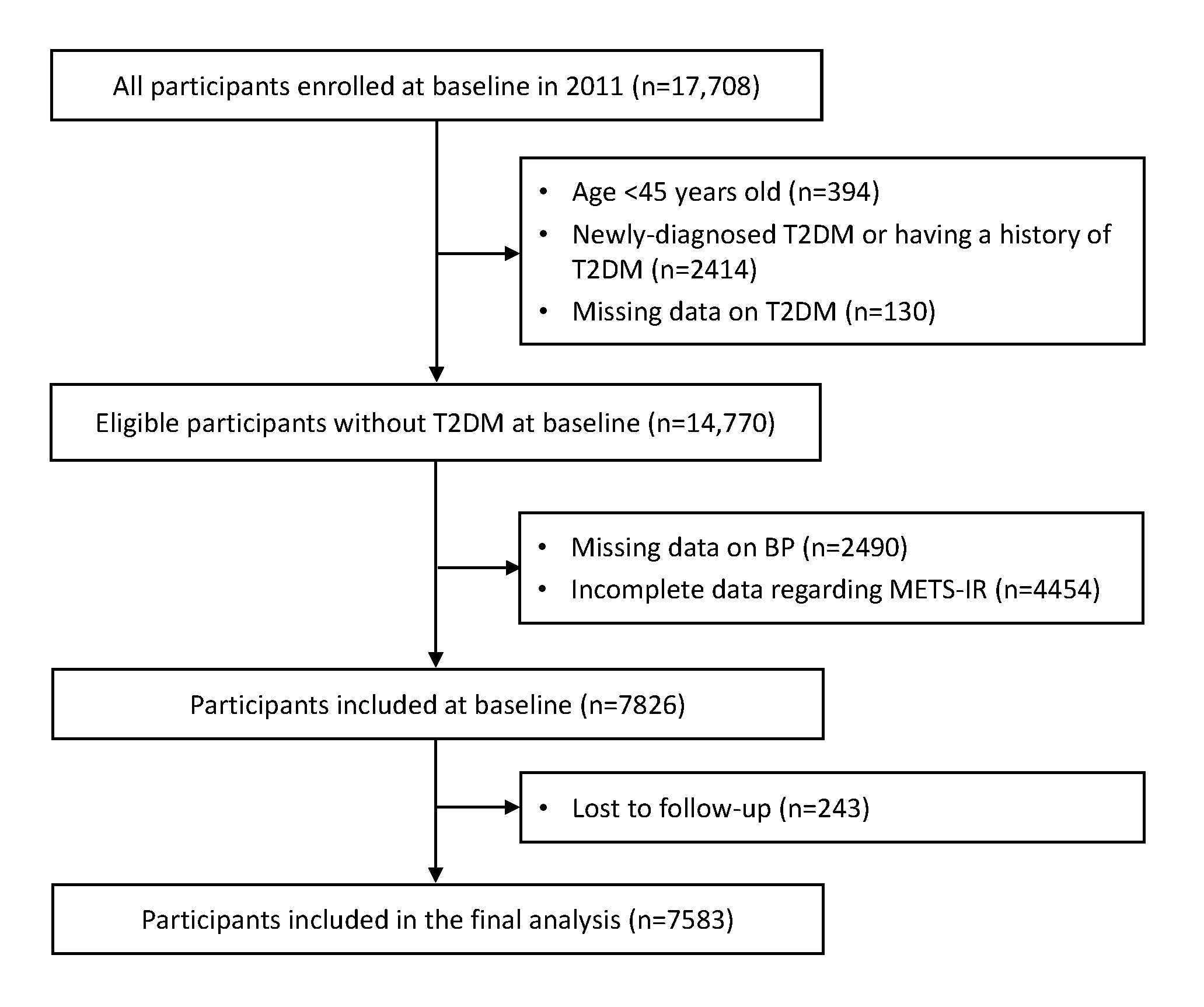


Note: T2DM, type 2 diabetes mellitus; BP, blood pressure; METS-IR, metabolic score for insulin resistance. The flowchart was adapted from the CONSORT flow diagram.

**Table S1 Clinical parameters of participants by BP at baseline and new-onset T2DM at follow-up**

| **Baseline parameters** | **Normal BP at baseline** | | |  | **Elevated BP at baseline** | | |
| --- | --- | --- | --- | --- | --- | --- | --- |
|  | **T2DM at follow-up (n = 116)** | **Non-T2DM at follow-up (n = 2,386)** | ***P* value** |  | **T2DM at follow-up (n = 411)** | **Non-T2DM at follow-up (n = 4,670)** | ***P* value** |
| **BMI^a^, kg/m^2^** | 23.1 (21.2, 25.4) | 22.0 (20.1, 24.2) | <.001 |  | 25.1 (22.6, 27.6) | 23.3 (21.0, 25.9) | <.001 |
| **WC^b^, cm** | 84.2±12.9 | 80.4±11.5 | <.001 |  | 89.1±13.7 | 84.8±12.3 | <.001 |
| **SBP^b^, mmHg** | 110.0±6.4 | 109.3±7.1 | .34 |  | 139.9±19.1 | 139.7±19.0 | .89 |
| **DBP^b^, mmHg** | 65.0±6.7 | 65.3±7.1 | .60 |  | 81.0±11.1 | 80.5±11.2 | .46 |
| **FPG^b^, mg/dL** | 104.3±13.0 | 97.9±11.5 | <.001 |  | 105.3±12.8 | 100.6±11.7 | <.001 |
| **TC^b^, mg/dL** | 190.7±36.1 | 187.8±35.1 | .38 |  | 199.1±37.6 | 194.2±38.2 | .01 |
| **TG^a^, mg/dL** | 105.8 (80.5, 160.2) | 92.0 (68.1, 129.2) | <.001 |  | 126.6 (86.7, 175.2) | 104.4 (74.3, 149.6) | <.001 |
| **HDL-C^a^, mg/dL** | 46.8 (35.2, 60.3) | 52.2 (42.9, 62.2) | <.001 |  | 46.8 (38.7, 55.3) | 49.9 (41.0, 60.7) | <.001 |
| **LDL-C^a^, mg/dL** | 116.2 (90.9, 134.7) | 112.1 (92.4, 133.8) | .64 |  | 119.1 (99.7, 143.0) | 115.2 (94.3, 138.8) | .02 |

Note: T2DM, type 2 diabetes mellitus; BMI, body mass index; WC, waist circumference; BP, blood pressure; SBP, systolic blood pressure; DBP, diastolic blood pressure; FPG, fasting plasma glucose; TC, total cholesterol; TG, triglycerides; HDL-C, high-density lipoprotein cholesterol; LDL-C, low-density lipoprotein cholesterol. ^a^Wilcoxon rank sum test was used. ^b^Two-sample *t*-test was used.

Data are presented as mean ± SD or median (interquartile range [IQR; 25^th^ to 75^th^ percentiles]) where appropriate. The two-sample *t*-test or the nonparametric Wilcoxon rank sum test, where appropriate, was used for between-group comparisons in participants with and without new-onset T2DM (i.e., T2DM at follow-up *vs.* non-T2DM at follow-up). Normal BP at baseline was defined as SBP <120 mmHg and DBP <80 mmHg. Elevated BP at baseline was defined as SBP ≥120 mmHg and/or DBP ≥80 mmHg or the presence of physician-diagnosed hypertension according to the 2018 Chinese Guidelines for the Management of Hypertension. To convert glucose values from mg/dL to mmol/L multiply by 0.0555. To convert total, HDL, and LDL cholesterol levels from mg/dL to mmol/L multiply by 0.02586. To convert triglyceride levels from mg/dL to mmol/L multiply by 0.01129.

**Table S2 Comparison of demographic characteristics between participants included in the final analysis and those excluded due to missing values**

| **Demographic characteristics** | **Participants included in the final analysis**  **(n = 7,583)** | **Participants excluded due to missing values (n = 7,317)** | ***P* value** |
| --- | --- | --- | --- |
| **Age, years** | 59.1±9.3 | 59.2±10.6 | .84 |
| **Sex** |  |  | <.001 |
| Male | 4,051 (53.4) | 3,572 (48.8) |  |
| Female | 3,532 (46.6) | 3,745 (51.2) | <.001 |
| **Place of residence** |  |  |  |
| Urban | 2,554 (33.7) | 3,369 (46.0) |  |
| Rural | 5,029 (66.3) | 3,948 (54.0) |  |
| **Education level** |  |  | <.001 |
| Elementary school or below | 5,326 (70.2) | 4,558 (62.5) |  |
| Middle school or above | 2,257 (29.8) | 2,736 (37.5) |  |
| **Cigarette smoking** |  |  | .011 |
| Current smoker | 2,317 (30.7) | 1,908 (28.7) |  |
| Non-smoker | 5,238 (69.3) | 4,738 (71.3) |  |
| **Alcohol drinking** |  |  | .15 |
| Regular drinker | 2,522 (33.3) | 2,478 (34.4) |  |
| Non-drinker | 5,056 (66.7) | 4,723 (65.6) |  |

Data are presented as n (%) or mean ± SD where appropriate. The two-sample *t*-test or the chi-square test, where appropriate, was used for comparison of demographic characteristics between participants included in the final analysis and those excluded due to missing values.

**Table S3 Sensitivity analysis by excluding participants who had new-onset T2DM within the first follow-up visit**

| **Variables** | **T2DM cases** | **Incidence rate^a^** | **Crude model** | |  | **Adjusted model** | |
| --- | --- | --- | --- | --- | --- | --- | --- |
|  |  |  | **HR (95%CI)** | ***P*** |  | **aHR (95%CI)** | ***P*** |
| **METS-IR, per *SD* unit increase** | |  |  |  |  |  |  |
| All participants at baseline | 391 | 8.2 | 1.43 (1.34, 1.54) | <.001 |  | 1.37 (1.25, 1.51) | <.001 |
| Participants with normal BP at baseline | 87 | 5.4 | 1.40 (1.24, 1.58) | <.001 |  | 1.45 (1.25, 1.69) | <.001 |
| Participants with elevated BP at baseline | 304 | 9.7 | 1.40 (1.28, 1.53) | <.001 |  | 1.33 (1.18, 1.49) | <.001 |
|  |  |  |  |  |  |  |  |
| **METS-IR, quartiles** |  |  |  |  |  |  |  |
| **All participants at baseline** |  |  |  |  |  |  |  |
| Quartile 1 (≤ 29.12) | 52 | 4.4 | 1.00 (reference) |  |  | 1.00 (reference) |  |
| Quartile 2 (29.13 to 33.40) | 64 | 5.3 | 1.20 (0.83, 1.72) | .34 |  | 1.18 (0.81, 1.71) | .40 |
| Quartile 3 (33.41 to 38.88) | 98 | 8.2 | 1.85 (1.32, 2.59) | <.001 |  | 1.76 (1.22, 2.53) | .002 |
| Quartile 4 (≥ 38.89) | 177 | 14.9 | 3.36 (2.46, 4.58) | <.001 |  | 3.02 (2.07, 4.41) | <.001 |
| *P* for trend |  |  | 1.08 (1.06, 1.10) | <.001 |  | 1.07 (1.05, 1.10) | <.001 |
| **Participants with normal BP at baseline** | |  |  |  |  |  |  |
| Quartile 1 (≤ 28.21) | 17 | 4.2 | 1.00 (reference) |  |  | 1.00 (reference) |  |
| Quartile 2 (28.22 to 31.64) | 10 | 2.5 | 0.58 (0.27, 1.27) | .18 |  | 0.65 (0.29, 1.45) | .30 |
| Quartile 3 (31.65 to 36.18) | 21 | 5.1 | 1.19 (0.63, 2.26) | .59 |  | 1.40 (0.70, 2.79) | .35 |
| Quartile 4 (≥ 36.19) | 39 | 9.9 | 2.34 (1.32, 4.14) | .003 |  | 2.60 (1.28, 5.29) | .008 |
| *P* for trend |  |  | 1.08 (1.04, 1.13) | <.001 |  | 1.09 (1.04, 1.14) | <.001 |
| **Participants with elevated BP at baseline** | |  |  |  |  |  |  |
| Quartile 1 (≤ 29.83) | 39 | 5.0 | 1.00 (reference) |  |  | 1.00 (reference) |  |
| Quartile 2 (29.84 to 34.48) | 52 | 6.6 | 1.30 (0.86, 1.96) | .22 |  | 1.25 (0.82, 1.91) | .30 |
| Quartile 3 (34.49 to 40.12) | 82 | 10.4 | 2.05 (1.40,3.01) | <.001 |  | 1.91 (1.26, 2.87) | .002 |
| Quartile 4 (≥ 40.13) | 131 | 16.4 | 3.22 (2.25, 4.61) | <.001 |  | 2.95 (1.92, 4.53) | <.001 |
| *P* for trend |  |  | 1.07 (1.05, 1.09) | <.001 |  | 1.07 (1.04, 1.09) | <.001 |

Note: METS-IR, metabolic score for insulin resistance; T2DM, type 2 diabetes mellitus.

Normal BP at baseline was defined as systolic BP <120 mmHg and diastolic BP <80 mmHg. Elevated BP at baseline was defined as systolic BP ≥120 mmHg and/or diastolic BP ≥80 mmHg or the presence of physician-diagnosed hypertension according to the 2018 Chinese Guidelines for the Management of Hypertension. Cox proportional hazard model adjusted for demographics, socioeconomic status, lifestyles, and anthropometric measurements. Tests for trend based on variables containing the median value for each quartile.

^a^ per 1000 person-years.

**Table S4 Sensitivity analysis by excluding participants with general obesity at baseline**

| **Variables** | **T2DM cases** | **Incidence rate^a^** | **Crude model** | |  | **Adjusted model** | |
| --- | --- | --- | --- | --- | --- | --- | --- |
|  |  |  | **HR (95%CI)** | ***P*** |  | **aHR (95%CI)** | ***P*** |
| **METS-IR, per *SD* unit increase** | |  |  |  |  |  |  |
| All participants at baseline | 313 | 7.3 | 1.48 (1.34, 1.63) | <.001 |  | 1.48 (1.32, 1.66) | <.001 |
| Participants with normal BP at baseline | 75 | 4.9 | 1.42 (1.17, 1.73) | <.001 |  | 1.60 (1.29, 1.98) | <.001 |
| Participants with elevated BP at baseline | 238 | 8.6 | 1.44 (1.28, 1.62) | <.001 |  | 1.42 (1.24, 1.63) | <.001 |
|  |  |  |  |  |  |  |  |
| **METS-IR, quartiles** |  |  |  |  |  |  |  |
| **All participants at baseline** |  |  |  |  |  |  |  |
| Quartile 1 (≤ 28.72) | 49 | 4.6 | 1.00 (reference) |  |  | 1.00 (reference) |  |
| Quartile 2 (28.73 to 32.51) | 54 | 5.0 | 1.06 (0.72, 1.56) | .76 |  | 1.09 (0.74, 1.62) | .66 |
| Quartile 3 (32.52 to 36.99) | 82 | 7.6 | 1.62 (1.14, 2.31) | .007 |  | 1.58 (1.08, 2.24) | .02 |
| Quartile 4 (≥ 37.00) | 128 | 12.0 | 2.57 (1.85, 3.57) | <.001 |  | 2.49 (1.68, 3.70) | <.001 |
| *P* for trend |  |  | 1.08 (1.05, 1.10) | <.001 |  | 1.08 (1.05, 1.10) | <.001 |
| **Participants with normal BP at baseline** | | |  |  |  |  |  |
| Quartile 1 (≤ 27.93) | 16 | 4.2 | 1.00 (reference) |  |  | 1.00 (reference) |  |
| Quartile 2 (27.94 to 31.28) | 10 | 2.6 | 0.63 (0.29, 1.39) | .25 |  | 0.75 (0.34, 1.69) | .49 |
| Quartile 3 (31.29 to 35.44) | 18 | 4.6 | 1.10 (0.56, 2.15) | .79 |  | 1.45 (0.70, 2.99) | .32 |
| Quartile 4 (≥ 35.45) | 31 | 8.2 | 1.96 (1.07, 3.59) | .03 |  | 2.75 (1.37, 5.52) | .005 |
| *P* for trend |  |  | 1.07 (1.02, 1.13) | .005 |  | 1.10 (1.04, 1.16) | <.001 |
| **Participants with elevated BP at baseline** | | |  |  |  |  |  |
| Quartile 1 (≤ 29.18) | 34 | 5.0 | 1.00 (reference) |  |  | 1.00 (reference) |  |
| Quartile 2 (29.19 to 33.17) | 43 | 6.2 | 1.21 (0.77, 1.90) | .40 |  | 1.17 (0.74, 1.85) | .51 |
| Quartile 3 (33.18 to 37.80) | 69 | 10.1 | 1.98 (1.31, 2.99) | .001 |  | 1.81 (1.16, 2.83) | .009 |
| Quartile 4 (≥ 37.81) | 92 | 13.2 | 2.59 (1.75, 3.84) | <.001 |  | 2.39 (1.51, 3.80) | <.001 |
| *P* for trend |  |  | 1.07 (1.05, 1.10) | <.001 |  | 1.07 (1.04, 1.10) | <.001 |

Note: METS-IR, metabolic score for insulin resistance; T2DM, type 2 diabetes mellitus.

General obesity at baseline was defined as BMI ≥28 kg/m^2^ at baseline according to the Working Group on Obesity in China. Normal BP at baseline was defined as systolic BP <120 mmHg and diastolic BP <80 mmHg. Elevated BP at baseline was defined as systolic BP ≥120 mmHg and/or diastolic BP ≥80 mmHg or the presence of physician-diagnosed hypertension according to the 2018 Chinese Guidelines for the Management of Hypertension. Cox proportional hazard model adjusted for demographics, socioeconomic status, lifestyles, and anthropometric measurements. Tests for trend based on variables containing the median value for each quartile.

^a^ per 1000 person-years.

**Table S5 Sensitivity analysis by supplementing incidence data using laboratory blood test results where data were available**

| **Variables** | **T2DM cases** | **Incidence rate^a^** | **Crude model** | |  | **Adjusted model** | |
| --- | --- | --- | --- | --- | --- | --- | --- |
|  |  |  | **HR (95%CI)** | ***P*** |  | **aHR (95%CI)** | ***P*** |
| **METS-IR, per *SD* unit increase** | |  |  |  |  |  |  |
| All participants at baseline | 873 | 18.9 | 1.34 (1.27, 1.41) | <.001 |  | 1.31 (1.22, 1.40) | <.001 |
| Participants with normal BP at baseline | 199 | 12.6 | 1.27 (1.14, 1.40) | <.001 |  | 1.27 (1.11, 1.44) | <.001 |
| Participants with elevated BP at baseline | 674 | 22.1 | 1.32 (1.23, 1.40) | <.001 |  | 1.32 (1.21, 1.43) | <.001 |
|  |  |  |  |  |  |  |  |
| **METS-IR, quartiles** |  |  |  |  |  |  |  |
| **All participants at baseline** |  |  |  |  |  |  |  |
| Quartile 1 (≤ 29.12) | 152 | 13.2 | 1.00 (reference) |  |  | 1.00 (reference) |  |
| Quartile 2 (29.13 to 33.40) | 155 | 13.2 | 1.00 (0.80, 1.25) | .98 |  | 1.04 (0.82, 1.30) | .77 |
| Quartile 3 (33.41 to 38.89) | 217 | 18.7 | 1.42 (1.16, 1.75) | <.001 |  | 1.45 (1.15, 1.82) | .001 |
| Quartile 4 (≥ 38.90) | 349 | 30.8 | 2.30 (1.90, 2.78) | <.001 |  | 2.23 (1.75, 2.84) | <.001 |
| *P* for trend |  |  | 1.06 (1.05, 1.06) | <.001 |  | 1.06 (1.04, 1.07) | <.001 |
| **Participants with normal BP at baseline** | | |  |  |  |  |  |
| Quartile 1 (≤ 28.21) | 45 | 11.5 | 1.00 (reference) |  |  | 1.00 (reference) |  |
| Quartile 2 (28.22 to 31.64) | 35 | 8.8 | 0.77 (0.50, 1.20) | .25 |  | 0.79 (0.50, 1.25) | .31 |
| Quartile 3 (31.65 to 36.18) | 48 | 11.9 | 1.04 (0.69, 1.56) | .86 |  | 1.05 (0.67, 1.65) | .84 |
| Quartile 4 (≥ 36.19) | 71 | 18.5 | 1.61 (1.11, 2.34) | .01 |  | 1.74 (1.14, 2.61) | .007 |
| *P* for trend |  |  | 1.04 (1.02, 1.07) | .001 |  | 1.04 (1.00, 1.07) | .04 |
| **Participants with elevated BP at baseline** | | |  |  |  |  |  |
| Quartile 1 (≤ 29.82) | 116 | 15.4 | 1.00 (reference) |  |  | 1.00 (reference) |  |
| Quartile 2 (29.83 to 34.47) | 126 | 16.4 | 1.067 (0.83, 1.37) | .62 |  | 1.10 (0.85, 1.42) | .47 |
| Quartile 3 (34.48 to 40.13) | 170 | 22.2 | 1.45 (1.15, 1.84) | .002 |  | 1.51 (1.17, 1.96) | .002 |
| Quartile 4 (≥ 40.14) | 262 | 34.6 | 2.22 (1.79, 2.76) | <.001 |  | 2.33 (1.78, 3.07) | <.001 |
| *P* for trend |  |  | 1.05 (1.04, 1.06) | <.001 |  | 1.06 (1.04, 1.07) | <.001 |

Note: METS-IR, metabolic score for insulin resistance; T2DM, type 2 diabetes mellitus.

The occurrence of new-onset T2DM was determined by a record of physician-diagnosed T2DM, or a laboratory blood test of FPG ≥126 mg/dL (7.0 mmol/L) or HbA1c ≥6.5% at follow-up. Normal BP at baseline was defined as systolic BP <120 mmHg and diastolic BP <80 mmHg. Elevated BP at baseline was defined as systolic BP ≥120 mmHg and/or diastolic BP ≥80 mmHg or the presence of physician-diagnosed hypertension according to the 2018 Chinese Guidelines for the Management of Hypertension. Cox proportional hazard model adjusted for demographics, socioeconomic status, lifestyles, and anthropometric measurements. Tests for trend based on variables containing the median value for each quartile.

^a^ per 1000 person-years.

**Table S6 Sensitivity analysis based on models fitted with time-varying covariates**

| **Variables** | **T2DM cases** | **Incidence rate^a^** | **Crude model** | |  | **Adjusted model** | |
| --- | --- | --- | --- | --- | --- | --- | --- |
|  |  |  | **HR (95%CI)** | ***P*** |  | **aHR (95%CI)** | ***P*** |
| **METS-IR, per *SD* unit increase** | |  |  |  |  |  |  |
| All participants at baseline | 527 | 11.0 | 1.44 (1.35, 1.53) | <.001 |  | 1.45 (1.35, 1.54) | <.001 |
| Participants with normal BP at baseline | 116 | 7.2 | 1.38 (1.23, 1.54) | <.001 |  | 1.45 (1.27, 1.66) | <.001 |
| Participants with elevated BP at baseline | 411 | 13.0 | 1.41 (1.31, 1.52) | <.001 |  | 1.39 (1.28, 1.51) | <.001 |
|  |  |  |  |  |  |  |  |
| **METS-IR, quartiles** |  |  |  |  |  |  |  |
| **All participants at baseline** |  |  |  |  |  |  |  |
| Quartile 1 (≤ 29.12) | 70 | 5.9 | 1.00 (reference) |  |  | 1.00 (reference) |  |
| Quartile 2 (29.13 to 33.40) | 87 | 7.2 | 1.22 (0.89, 1.67) | .22 |  | 1.25 (0.91, 1.72) | .17 |
| Quartile 3 (33.41 to 38.89) | 135 | 11.3 | 1.93 (1.44, 2.57) | <.001 |  | 2.00 (1.49, 2.68) | <.001 |
| Quartile 4 (≥ 38.90) | 235 | 19.9 | 3.39 (2.60, 4.43) | <.001 |  | 3.51 (2.65, 4.65) | <.001 |
| *P* for trend |  |  | 1.08 (1.07, 1.10) | <.001 |  | 1.08 (1.07, 1.10) | <.001 |
| **Participants with normal BP at baseline** | | |  |  |  |  |  |
| Quartile 1 (≤ 28.21) | 22 | 5.5 | 1.00 (reference) |  |  | 1.00 (reference) |  |
| Quartile 2 (28.22 to 31.64) | 13 | 3.2 | 0.59 (0.30, 1.16) | .13 |  | 1.34 (0.75, 1.39) | .33 |
| Quartile 3 (31.65 to 36.18) | 33 | 8.1 | 1.48 (0.86, 2.53) | .16 |  | 2.10 (1.18, 3.73) | .01 |
| Quartile 4 (≥ 36.19) | 48 | 12.1 | 2.22 (1.34, 3.68) | .002 |  | 3.91 (2.06, 7.39) | <.001 |
| *P* for trend |  |  | 1.08 (1.04, 1.12) | <.001 |  | 1.09 (1.04, 1.13) | <.001 |
| **Participants with elevated BP at baseline** | | |  |  |  |  |  |
| Quartile 1 (≤ 29.82) | 55 | 7.0 | 1.00 (reference) |  |  | 1.00 (reference) |  |
| Quartile 2 (29.83 to 34.47) | 70 | 8.8 | 1.25 (0.88, 1.77) | .22 |  | 1.19 (0.81, 1.74) | .38 |
| Quartile 3 (34.48 to 40.13) | 110 | 13.9 | 1.98 (1.43, 2.73) | <.001 |  | 1.84 (1.29, 2.62) | <.001 |
| Quartile 4 (≥ 40.14) | 176 | 22.2 | 3.17 (2.34, 4.28) | <.001 |  | 3.00 (2.14, 4.19) | <.001 |
| *P* for trend |  |  | 1.07 (1.06, 1.09) | <.001 |  | 1.07 (1.05, 1.09) | <.001 |

Note: METS-IR, metabolic score for insulin resistance; T2DM, type 2 diabetes mellitus.

The occurrence of new-onset T2DM was determined by a record of physician-diagnosed T2DM, or a laboratory blood test of FPG ≥126 mg/dL (7.0 mmol/L) or HbA1c ≥6.5% at follow-up. Normal BP was defined as systolic BP <120 mmHg and diastolic BP <80 mmHg. Elevated BP was defined as systolic BP ≥120 mmHg and/or diastolic BP ≥80 mmHg or the presence of physician-diagnosed hypertension. Cox proportional hazard model adjusted for sex, place of residence, education level, and time-varying covariates including age, annual household income, living relationships, cigarette smoking, alcohol drinking, blood pressure, and waist circumference. Tests for trend based on variables containing the median value for each quartile. The last observation carried forward (LOCF) imputation method was used to take into account missing values where applicable.

^a^ per 1000 person-years.

**Table S7 Predictive capability of baseline METS-IR on top of blood glucose for new-onset T2DM**

|  | **AUC (95%CI)** | **NRI (95%CI)** | **IDI (95%CI)** | **Cutoff points** | **Youden index** |
| --- | --- | --- | --- | --- | --- |
| **All participants** |  |  |  |  |  |
| Baseline model | 0.640 (0.615, 0.665)^a^ | Reference | Reference | – | – |
| Baseline model + FPG | 0.685 (0.659, 0.710)^a^ | 0.388 (0.302, 0.476)^a^ | 0.016 (0.013, 0.020)^a^ | – | – |
| Baseline model + FPG + METS-IR | 0.692 (0.667, 0.716)^a^ | 0.432 (0.345, 0.519)^a^ | 0.018 (0.015, 0.022)^a^ | 35.33 | 0.24 |
| **Participants with normal BP** |  |  |  |  |  |
| Baseline model | 0.643 (0.591, 0.695)^a^ | Reference | Reference | – | – |
| Baseline model + FPG | 0.697 (0.645, 0.749)^a^ | 0.418 (0.234, 0.601)^a^ | 0.018 (0.011, 0.026)^a^ | – | – |
| Baseline model + FPG + METS-IR | 0.708 (0.659, 0.758)^a^ | 0.474 (0.291, 0.657)^a^ | 0.026 (0.017, 0.036)^a^ | 31.69 | 0.22 |
| **Participants with elevated BP** |  |  |  |  |  |
| Baseline model | 0.633 (0.604, 0.662)^a^ | Reference | Reference | – | – |
| Baseline model + FPG | 0.675 (0.645, 0.704)^a^ | 0.369 (0.270, 0.469)^a^ | 0.015 (0.012, 0.019)^a^ | – | – |
| Baseline model + FPG + METS-IR | 0.680 (0.651, 0.709)^a^ | 0.421 (0.322, 0.520)^a^ | 0.016 (0.012, 0.021)^a^ | 35.47 | 0.23 |

^a^*P*<.001

Note: METS-IR, metabolic score for insulin resistance; T2DM, type 2 diabetes mellitus; FPG, fasting plasma glucose; AUC, area under receiver operating characteristic (ROC) curve; NRI, net reclassification improvement; IDI, integrated discrimination improvement. Normal BP at baseline was defined as systolic BP <120 mmHg and diastolic BP <80 mmHg. Elevated BP at baseline was defined as systolic BP ≥120 mmHg and/or diastolic BP ≥80 mmHg or the presence of physician-diagnosed hypertension according to the 2018 Chinese Guidelines for the Management of Hypertension. The baseline model included age, sex, place of residence, education level, annual household income, living relationships, cigarette smoking, alcohol drinking, blood pressure, and waist circumference.
